# Reflux Grade Is Associated with Postoperative Outcomes After Ureteral Reimplantation in Children: A Retrospective Cohort Study from a Middle-Income Healthcare Setting

**DOI:** 10.3390/children13070869

**Published:** 2026-06-29

**Authors:** Andrea Canelos-Dueñas, Fabricio González-Andrade

**Affiliations:** 1 School of Medical Specialties, College of Health Sciences, San Francisco de Quito University USFQ, Diego de Robles Street s/n y Pampite, Quito 170901, Ecuador; acanelosd@alumni.usfq.edu.ec; 2 Dean’s Office of Postgraduate Studies in Health, Faculty of Health and Wellness Sciences, Indoamerica Technological University, Machala Street y Sabanilla Street, Quito 170301, Ecuador

**Keywords:** vesicoureteral reflux, ureteral reimplantation, reflux severity, laparoscopic surgery, open surgery, pediatric urology, surgical outcomes, Ecuador, recurrence, renal preservation, Sustainable Development Goal 3

## Abstract

**Highlights:**

**What are the main findings?**

- Overall reflux resolution was achieved in 68 of 90 patients (75.6%), recurrence occurred in 12 patients (13.3%), and reintervention was required in 8 patients (8.9%).
- Grade V VUR was associated with lower odds of reflux resolution and higher odds of recurrence compared with Grade III VUR, but these estimates were imprecise because the Grade V subgroup was small and recurrence events were few.
- Resolution rates were similar between open and laparoscopic surgery, while recurrence was numerically lower after laparoscopy without statistical significance.

**What are the implications of the main findings?**

- VUR grade may be useful as one marker for postoperative risk stratification in children undergoing ureteral reimplantation, but it should not be interpreted as the sole determinant of surgical outcome.
- Children with Grade V VUR may require closer postoperative surveillance and careful counseling regarding recurrence risk, while considering reflux etiology, bladder function, renal status, and follow-up intensity.
- Comparisons between surgical approaches should account for selection bias, surgeon experience, equipment availability, reflux etiology, bladder dysfunction, renal status, operative time, and follow-up duration.
- Prospective multicenter studies with standardized imaging, renal functional follow-up, uniform outcome definitions, complete missing-data reporting, and adequate event numbers are needed to confirm these findings.

**Abstract:**

Background: Vesicoureteral reflux (VUR) is a common pediatric urologic disorder associated with recurrent febrile urinary tract infections, renal scarring, hypertension, and potential long-term renal morbidity. Postoperative outcomes after ureteral reimplantation may be influenced by reflux grade, reflux etiology, bladder function, renal status, operative technique, surgeon experience, and follow-up intensity. However, real-world evidence from low- and middle-income healthcare settings remains limited, particularly for surgically treated children with high-grade VUR. Objective: To describe postoperative outcomes after ureteral reimplantation in children with Grade III–V VUR and to explore the association of VUR grade and surgical approach with reflux resolution, recurrence, and reintervention in a surgically selected pediatric cohort. Methods: We conducted a retrospective cohort study at a tertiary pediatric referral center in Ecuador. The study included 90 children aged 0 to 15 years with Grade III–V VUR confirmed by voiding cystourethrography who underwent open or laparoscopic ureteral reimplantation between January 2019 and January 2024. Demographic, clinical, imaging, and surgical variables were extracted from medical records. Postoperative outcomes included reflux resolution, recurrence, and reintervention. Logistic regression models were used as exploratory analyses only. Because the cohort included only operated patients, treatment allocation was nonrandomized, event numbers were limited, and several relevant prognostic variables were incompletely documented, adjusted estimates were interpreted cautiously and were not used to infer causality. Results: Overall reflux resolution was achieved in 68 of 90 patients (75.6%), recurrence occurred in 12 patients (13.3%), and reintervention was required in 8 patients (8.9%). Resolution rates were similar after open and laparoscopic surgery (44/59, 74.6% vs. 24/31, 77.4%; *p* = 0.766). Recurrence was numerically lower after laparoscopic than open reimplantation, but the difference was not statistically significant (2/31, 6.5% vs. 10/59, 16.9%; *p* = 0.164). Reintervention rates were also similar between groups (3/31, 9.7% vs. 5/59, 8.5%; *p* = 0.849). In exploratory multivariable analysis, Grade V VUR was associated with lower odds of reflux resolution (OR, 0.06; 95% CI, 0.01–0.40; *p* = 0.003) and higher odds of recurrence (OR, 16.69; 95% CI, 1.88–148.32; *p* = 0.012) compared with Grade III VUR. Surgical approach was not independently associated with resolution, recurrence, or reintervention. The small Grade V subgroup, the limited number of recurrence and reintervention events, and the wide confidence intervals indicate substantial statistical imprecision. Conclusions: In this surgically treated pediatric cohort from a tertiary referral center in Ecuador, ureteral reimplantation was associated with reflux resolution in approximately three-quarters of patients. Higher reflux grade, particularly Grade V disease, was associated with less favorable postoperative outcomes in exploratory analyses, but these findings should not be interpreted as causal or definitive because of the small subgroup size, limited event numbers, selection bias, and incomplete documentation of reflux etiology, bladder dysfunction, renal scarring, renal function, surgeon experience, and follow-up duration. Open and laparoscopic approaches showed comparable resolution and reintervention rates, while the lower recurrence observed after laparoscopy did not reach statistical significance. Future prospective studies should standardize outcome definitions, distinguish imaging-confirmed from clinically documented resolution, report follow-up duration, and account for reflux etiology, bladder function, renal status, surgical experience, and healthcare access.

## 1. Introduction

Vesicoureteral reflux (VUR) is one of the most common congenital anomalies of the urinary tract in children and is defined as the retrograde passage of urine from the bladder into the ureter and renal collecting system [1]. Its clinical relevance lies in its association with recurrent febrile urinary tract infections, renal cortical scarring, hypertension, and selected cases of long-term renal morbidity [2]. Although many children with low-grade reflux experience spontaneous resolution, high-grade VUR is less likely to resolve and is more frequently associated with persistent reflux, recurrent infection, and renal injury [3]. For this reason, early identification of children at increased risk of persistent or complicated reflux remains a central objective in pediatric nephrology and urology [4]. The prognosis of VUR depends not only on anatomical grade but also on patient age, laterality, renal morphology, bladder function, infection history, and associated urinary tract abnormalities [5]. Therefore, contemporary management requires individualized risk stratification rather than a uniform treatment strategy for all children with VUR [6].

VUR is traditionally classified as primary or secondary, and this distinction is essential because these entities differ in pathophysiology, treatment indications, surgical planning, and prognosis [7]. Primary VUR usually results from congenital incompetence of the ureterovesical junction, whereas secondary VUR occurs in association with elevated bladder pressure, abnormal voiding dynamics, obstructive uropathy, neurogenic bladder, posterior urethral valves, bladder-bowel dysfunction, or other functional and anatomical conditions [8]. Because reflux etiology may influence recurrence, reintervention, and long-term renal outcomes, reflux grade and reflux etiology should be interpreted as clinically distinct variables when postoperative outcomes are analyzed [9]. Failure to account for underlying etiology, bladder dysfunction, renal scarring, renal function, and follow-up intensity may introduce residual confounding, particularly when high-grade reflux is evaluated as the main prognostic marker [10]. Thus, studies of surgical outcomes should avoid implying that reflux severity alone determines prognosis when important clinical and etiological variables are incompletely documented [11].

The diagnosis and follow-up of VUR rely on complementary imaging modalities that provide anatomical and functional information [12]. Voiding cystourethrography remains the reference standard for confirming VUR and assigning reflux grade according to the degree of retrograde filling and upper urinary tract dilation [13]. Renal ultrasonography is widely used as a first-line, noninvasive examination to evaluate hydronephrosis, renal size, cortical morphology, ureteral dilation, and postoperative changes [14]. Dimercaptosuccinic acid scintigraphy provides additional information on renal cortical defects and differential renal function, particularly in children with febrile urinary tract infections or suspected reflux nephropathy [15]. More recently, contrast-enhanced voiding urosonography has emerged as a radiation-sparing alternative for diagnosis and surveillance in selected pediatric patients [16]. Together, these tools help define baseline renal status, guide treatment decisions, and assess whether surgical correction is associated with preservation of renal morphology and function [17].

Management strategies for pediatric VUR include surveillance, continuous antibiotic prophylaxis, endoscopic injection, open ureteral reimplantation, and minimally invasive reconstructive approaches [18]. Continuous antibiotic prophylaxis may reduce recurrent febrile urinary tract infections in selected children, but it does not eliminate the need for careful monitoring of renal status, bladder function, adherence, and breakthrough infections [19]. Endoscopic treatment has become an important option in contemporary VUR algorithms because it offers lower invasiveness and may achieve favorable outcomes in selected intermediate- and high-grade primary reflux cases [20]. Open ureteral reimplantation, including intravesical and extravesical techniques, remains a highly effective standard treatment, particularly for persistent high-grade reflux, recurrent infections, renal scarring, or failed conservative management [21]. Laparoscopic and robotic-assisted reimplantation have expanded the reconstructive options available for children, although outcomes may depend on surgical expertise, patient selection, equipment availability, institutional resources, and case complexity [22]. Consequently, comparisons between open and minimally invasive approaches should be interpreted with caution when treatment allocation is not randomized and when surgeon experience or technical availability varies across patients [23].

In low- and middle-income healthcare settings, the diagnosis and treatment of high-grade VUR may be influenced by delayed referral, limited access to pediatric subspecialists, variable availability of diagnostic imaging, restricted access to minimally invasive equipment, and inconsistent postoperative follow-up [24]. These system-level factors can affect the timing of diagnosis, the choice of surgical approach, the feasibility of standardized imaging surveillance, and the completeness of postoperative outcome assessment [25]. Geographic barriers and unequal access to specialized pediatric surgical services may further influence referral patterns, disease severity at presentation, and adherence to postoperative surveillance [26]. In such contexts, open surgery may remain the most accessible reconstructive option, whereas laparoscopy may be reserved for selected patients depending on institutional capacity, trained personnel, equipment availability, and operating room conditions [27]. However, limited resources do not reduce the importance of documenting reflux etiology, bladder dysfunction, renal imaging findings, surgical indications, operative technique, surgeon experience, and follow-up duration [28]. Data from underrepresented pediatric populations are clinically valuable, but interpretation must acknowledge selection bias, information bias, residual confounding, and limited statistical power when outcome events are few [29].

This study evaluated postoperative outcomes after ureteral reimplantation in Ecuadorian children with high-grade VUR treated at a tertiary pediatric referral center. The primary objective was to assess the association between reflux grade and postoperative outcomes, including reflux resolution, recurrence, and reintervention, without implying a causal relationship between anatomical severity and surgical prognosis. A secondary objective was to compare outcomes between open and laparoscopic reimplantation while considering potential selection bias, limited sample size, nonrandomized treatment allocation, and resource-dependent surgical decision-making. Because the cohort included only surgically treated patients, the findings should be interpreted as applicable to a selected operative population rather than to all children with Grade III–V VUR. The study also aimed to contextualize its findings within the broader challenges of VUR care in a middle-income healthcare setting, where diagnostic access, referral pathways, surgical resources, and follow-up intensity may influence observed outcomes. By emphasizing disease severity, surgical approach, healthcare access, and methodological limitations, this study contributes real-world evidence from an underrepresented pediatric population while recognizing that reflux etiology, bladder dysfunction, renal status, surgeon experience, and standardized follow-up remain essential determinants of long-term outcomes.

## 2. Methods

### 2.1. Study Design

This was a retrospective, observational cohort study based on routinely collected clinical data from pediatric patients with high-grade vesicoureteral reflux (VUR) who underwent ureteral reimplantation. The study was designed to describe postoperative outcomes and to explore associations between clinical, anatomical, and surgical variables and postoperative reflux resolution, recurrence, and reintervention. The manuscript was prepared in accordance with the Strengthening the Reporting of Observational Studies in Epidemiology (STROBE) recommendations for observational studies [23]. Because this was a retrospective study, no intervention was performed for research purposes, and all diagnostic, therapeutic, surgical, and follow-up decisions had been made by the treating pediatric urology team before data extraction.

### 2.2. Setting

The study was conducted at Baca Ortiz Pediatric Hospital, a tertiary pediatric referral center located in Quito, Ecuador. The hospital receives children from urban and rural areas across the country and functions as a national referral center for pediatric urology. Patients are commonly referred for evaluation after recurrent febrile urinary tract infections, prenatal or postnatal hydronephrosis, abnormal urinary tract imaging, suspected renal damage, persistent high-grade VUR, or failure of conservative management. Surgical correction was performed between January 2019 and January 2024.

### 2.3. Participants and Eligibility Criteria

Eligible patients were identified through institutional surgical records and electronic and physical medical charts. The cohort included children aged 0 to 15 years with Grade III, IV, or V VUR confirmed by voiding cystourethrography (VCUG), who underwent open or laparoscopic ureteral reimplantation during the study period. All available patients fulfilling the eligibility criteria were reviewed. The study population, therefore, represents a surgically treated cohort and does not include children with VUR who were managed conservatively during the same period. Inclusion criteria were age 15 years or younger at the time of surgery, diagnosis of Grade III–V VUR confirmed by VCUG, surgical correction by open or laparoscopic ureteral reimplantation, and availability of sufficient preoperative and postoperative information to classify the main outcome. Exclusion criteria were age older than 15 years, absence of postoperative outcome data, renal disease unrelated to VUR, previous major urinary tract reconstruction before the index procedure, neurological disorders causing neurogenic bladder when these prevented standardized interpretation of reflux outcomes, and complex congenital urinary tract anomalies in which reflux outcome could not be reliably attributed to ureteral reimplantation.

### 2.4. Classification of VUR Grade, Etiology, and Associated Conditions

VUR grade was assigned according to VCUG findings and categorized as Grade III, Grade IV, or Grade V. Reflux laterality was recorded as left-sided, right-sided, or bilateral. VUR etiology was classified as primary, secondary, or not documented, according to information available in the clinical record. Primary VUR was defined as reflux associated with congenital incompetence of the ureterovesical junction in the absence of an identifiable obstructive, neurological, or functional bladder disorder. Secondary VUR was defined as reflux occurring in association with elevated bladder pressure, bladder dysfunction, obstructive uropathy, neurological disease, or other anatomical or functional abnormalities that could alter voiding dynamics or ureterovesical competence. The following associated diagnoses were reviewed when recorded: posterior urethral valves, duplex collecting system, ureterocele, neurogenic bladder, bladder-bowel dysfunction, dysfunctional voiding, obstructive uropathy, and other congenital urinary tract anomalies. Reflux grade and reflux etiology were treated as distinct variables. When etiology or associated conditions were not sufficiently documented, these variables were classified as missing or not documented rather than inferred.

### 2.5. Indications for Surgical Treatment

Surgical treatment was indicated according to institutional pediatric urology criteria and individualized clinical judgment. Main indications included persistent Grade III–V VUR, recurrent febrile urinary tract infections despite medical management, breakthrough infections during continuous antibiotic prophylaxis, renal scarring or abnormal renal morphology on imaging, progressive hydronephrosis or upper urinary tract dilation, failure of conservative management, and concern for ongoing renal damage. The decision to proceed with surgery was made by the treating pediatric urology team after reviewing clinical history, infection recurrence, VCUG findings, renal ultrasound findings, renal functional imaging when available, patient age, reflux grade, reflux laterality, suspected bladder dysfunction, anatomical complexity, and family adherence to follow-up. Because the study did not include conservatively treated children, the analysis was restricted to operated patients and was not designed to compare surgical treatment with non-surgical management.

### 2.6. Selection of Surgical Approach

The choice between open and laparoscopic ureteral reimplantation was not randomized. Surgical approach was selected by the treating surgeon according to patient characteristics, reflux grade, laterality, anatomical complexity, suspected associated anomalies, previous clinical course, equipment availability, operating room resources, availability of trained personnel, and surgeon experience. When information was available, the reason for selecting open or laparoscopic surgery was extracted from operative notes, preoperative assessments, and institutional records. Open ureteral reimplantation was available throughout the study period and represented the established reconstructive approach at the institution. Laparoscopic reimplantation was performed in selected patients when pediatric laparoscopic instruments, trained personnel, and operating room conditions were available. Because treatment allocation depended on clinical and institutional factors, comparisons between open and laparoscopic reimplantation were considered nonrandomized comparisons within a selected operative cohort.

### 2.7. Surgical Techniques and Operator-Related Variables

Open ureteral reimplantation included standard antireflux reconstructive techniques performed according to intraoperative anatomy and surgeon preference. When specified in the operative report, the technique was recorded as intravesical, including Cohen cross-trigonal reimplantation or Politano-Leadbetter repair, or extravesical, including Lich-Gregoir reimplantation. Laparoscopic ureteral reimplantation was classified according to the operative route documented in the surgical report, such as transperitoneal, extravesical laparoscopic, or pneumovesicoscopic. If the laparoscopic route was not specified, the case was recorded as laparoscopic reimplantation not otherwise specified. Operative variables included surgical approach, laterality of repair, operative technique when documented, operative time, technical conversion, intraoperative complications, and other procedure-related adverse events. Surgeon experience, number of participating surgeons, and period of institutional experience with laparoscopy were reviewed when documented. When these variables were unavailable or inconsistently recorded, they were classified as missing and were not included in adjusted models.

### 2.8. Preoperative Assessment

Preoperative assessment included demographic, clinical, laboratory, and imaging data obtained from electronic and physical medical records. Demographic variables included age, sex, ethnicity, region of residence, and age at surgery. Clinical variables included gestational age, history of febrile urinary tract infection, dysuria, prenatal ultrasound findings, previous conservative management, continuous antibiotic prophylaxis when documented, breakthrough infections, and clinical suspicion or documentation of bladder-bowel dysfunction or dysfunctional voiding. Imaging variables included VUR grade and laterality on VCUG, renal ultrasound findings, hydronephrosis severity, ureteral dilation, renal size, cortical morphology, and associated urinary tract abnormalities. When available, dimercaptosuccinic acid scintigraphy findings were reviewed to identify renal cortical defects, renal scarring, and differential renal function. Renal function tests and additional imaging studies were recorded when available.

### 2.9. Postoperative Follow-Up

Postoperative follow-up was based on outpatient pediatric urology visits, clinical evaluation, and imaging studies performed according to institutional practice and clinical indication. Follow-up information was extracted from the date of surgery to the last available postoperative visit. Follow-up duration was calculated in months when the date of the index surgery and the date of the last postoperative assessment were both available. The number and proportion of patients with available follow-up duration data were recorded, and missing follow-up duration was reported separately. Postoperative assessment included documentation of febrile urinary tract infections, symptoms suggestive of recurrent reflux, renal ultrasound findings, VCUG findings when performed, additional imaging studies when available, and the need for further intervention. When postoperative imaging was not available, the basis for outcome classification was recorded as specialist documentation rather than imaging-confirmed resolution. The proportion of resolved cases confirmed by imaging and the proportion classified by clinical documentation alone were reported separately when available.

### 2.10. Outcome Definitions

The primary outcome was reflux resolution. Resolution was defined as the absence of VUR on postoperative imaging, preferably VCUG. When postoperative VCUG was not available, resolution could also be classified if the treating pediatric urologist clearly documented reflux resolution based on available postoperative follow-up assessment. Because these two sources are not equivalent, outcome classification was stratified as imaging-confirmed resolution or clinically documented resolution without confirmatory postoperative VCUG. Reflux recurrence was defined as reappearance of VUR after documented postoperative resolution, persistent clinically significant reflux after surgical correction, or recurrent reflux documented by postoperative imaging or specialist assessment. Reintervention was defined as the need for any additional surgical or endoscopic procedure to treat persistent, recurrent, or clinically significant VUR after the index ureteral reimplantation. Intraoperative complications were defined as any adverse event recorded during the surgical procedure, including technical conversion to open surgery, bladder injury, ureteral injury, vascular injury, anesthetic or positioning-related complications, and other procedure-related events. Technical conversion was defined as planned laparoscopic surgery converted to open surgery during the same operative session. Equipment-related limitations included documented problems with availability, function, or adequacy of pediatric laparoscopic instruments, optical systems, insufflation, operative exposure, or other operating room resources. Technical limitations included inadequate visualization, difficult dissection, anatomical complexity, inability to complete ureteral mobilization or tunneling safely, or intraoperative concern for patient safety. When the specific complication or limitation was not described in the operative note, the event was recorded as unspecified.

### 2.11. Data Sources and Data Management

Data were obtained from electronic and physical medical records using a structured data extraction form. A single investigator performed the initial extraction to improve consistency and reduce measurement variability. Extracted variables included demographic characteristics, clinical presentation, VUR grade, reflux laterality, suspected reflux etiology, associated urinary tract conditions, imaging findings, surgical indication, surgical approach, operative technique when available, operative time, intraoperative complications, postoperative follow-up, reflux resolution, recurrence, and reintervention. All personal identifiers were removed before analysis, and the working dataset was anonymized. For each variable, the number of available observations and missing values was recorded. Variables with incomplete information were analyzed using the available denominator for that variable. No statistical imputation was performed. A supplementary missing-data table was prepared to report variable-level missingness for clinically relevant variables, including reflux etiology, bladder dysfunction, DMSA findings, renal scarring, renal function, surgeon experience, operative technique, follow-up duration, imaging-confirmed resolution, recurrence, and reintervention.

### 2.12. Bias and Confounding

The study considered several potential sources of bias during design, analysis, and interpretation. Selection bias may have occurred because the cohort included only surgically treated children and excluded patients managed conservatively during the same period. Confounding by indication may have affected comparisons between open and laparoscopic reimplantation because the surgical approach was selected according to clinical complexity, anatomical characteristics, institutional resources, equipment availability, and surgeon experience rather than random allocation. Information bias may have resulted from retrospective data collection, incomplete documentation of reflux etiology, variable evaluation of bladder dysfunction, incomplete DMSA or renal functional data, and non-standardized postoperative imaging. Residual confounding may have persisted because some clinically relevant variables, including bladder-bowel dysfunction, ureteral remodeling, surgeon experience, adherence to antibiotic prophylaxis, socioeconomic factors, and adherence to follow-up, were not consistently available. Follow-up duration was specifically considered because reflux resolution, recurrence, and reintervention are time-dependent outcomes.

### 2.13. Study Size

The study size was determined by the number of eligible patients who underwent surgical correction for high-grade VUR during the study period. No formal a priori sample size calculation or power analysis was performed because this was a retrospective cohort based on available institutional records. A total of 90 patients met eligibility criteria and were included in the analysis. The number of outcome events was limited, particularly for recurrence and reintervention. Therefore, statistical power was restricted, and adjusted regression models were considered exploratory and hypothesis-generating. The number of events for each outcome was explicitly reported before presenting regression analyses.

### 2.14. Statistical Analysis

Descriptive statistics were used to summarize demographic, clinical, imaging, surgical, and postoperative characteristics. Categorical variables were expressed as frequencies and percentages. Continuous variables were summarized as mean and standard deviation or median and interquartile range, according to data distribution. Normality was assessed before selecting parametric or nonparametric tests. Comparisons between open and laparoscopic groups were performed using the chi-square test or Fisher’s exact test for categorical variables, as appropriate. For continuous variables, Student’s *t* test or the Mann–Whitney U test was used according to normality and variance assumptions.

Operative time was reported by surgical approach using central tendency and dispersion measures when continuous operative time values were available. If only categorical operative time were available, operative time was summarized using the recorded categories and compared between groups using categorical methods. Because pneumovesicoscopic reimplantation was not performed or not documented as a distinct operative category in the analyzed dataset, laparoscopic cases were reported according to the available operative classification.

Logistic regression models were used only as exploratory analyses to examine associations between selected predictors and postoperative outcomes, including reflux resolution, recurrence, and reintervention. Predictor variables were limited to sex, age group, surgical approach, and VUR grade. Clinically relevant variables such as reflux etiology, bilateral disease, bladder dysfunction, renal abnormalities, DMSA findings, surgeon experience, and follow-up duration were not included in adjusted models when documentation was incomplete or when event numbers were insufficient. Because only a small number of recurrence and reintervention events were observed, regression estimates for these outcomes were interpreted as statistically unstable and hypothesis-generating rather than confirmatory.

Odds ratios with 95% confidence intervals were reported. Wide confidence intervals were interpreted as evidence of statistical imprecision. Regression outputs were not used to infer causality or to establish definitive superiority of any surgical approach. For Grade V VUR, the absolute number of patients and the absolute number of resolution, recurrence, and reintervention events were reported to contextualize regression estimates derived from this small subgroup. A *p*-value less than 0.05 was considered statistically significant for descriptive comparisons, but emphasis was placed on effect size, confidence intervals, event numbers, and clinical interpretability. All analyses were performed using SPSS version 22.0.

### 2.15. Ethical Considerations

The study was approved by the Research Ethics Committee on Human Subjects of Universidad San Francisco de Quito (protocol No. 100-2024-CA24028TPG-CEISH-USFQ; approved on 13 May 2024). As the study was retrospective, observational, and based on anonymized medical record data, informed consent was waived by the ethics committee. No identifiable patient information was included in the analysis or manuscript.

## 3. Results

**Figure 1:**
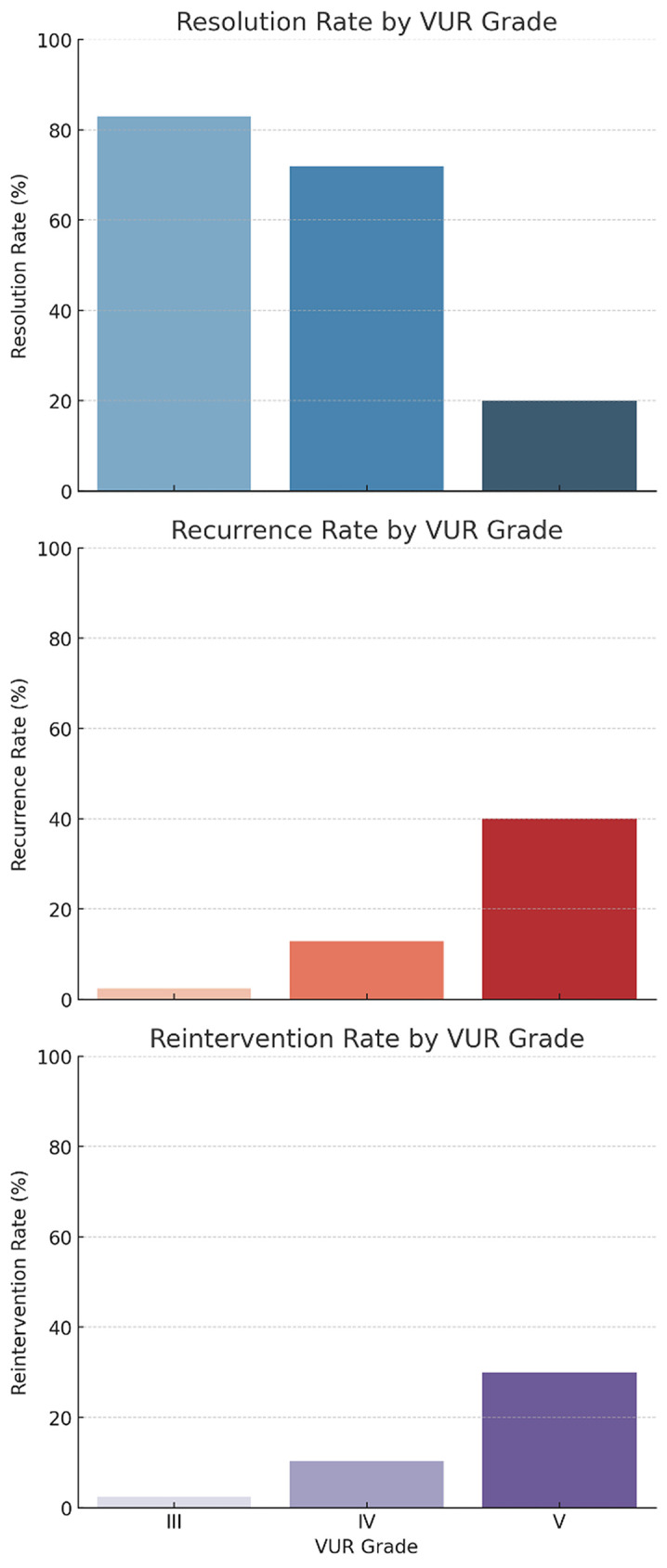
Surgical outcomes by VUR grade in pediatric patients. This vertical composite figure shows postoperative outcomes according to vesicoureteral reflux (VUR) grade (III, IV, and V). The top panel shows reflux resolution, the middle panel shows recurrence, and the bottom panel shows reintervention. Resolution rates decreased with increasing VUR grade, with the highest resolution observed in Grade III, an intermediate rate in Grade IV, and the lowest rate in Grade V. Conversely, recurrence and reintervention rates increased across higher VUR grades. These patterns suggest an association between greater anatomical severity and less favorable postoperative outcomes. However, findings for Grade V should be interpreted cautiously because of the small subgroup size, retrospective study design, and potential residual confounding. Overall, the figure supports severity-based postoperative risk stratification and closer follow-up for children with high-grade VUR.

Table 1 presents the distribution of patients by therapeutic management according to demographic characteristics. Table 2 presents the distribution according to clinical characteristics, while Table 3 presents the distribution according to surgical characteristics. Table 4, Table 5 and Table 6 report the multivariate analyses used to predict reflux resolution, reflux recurrence, and reintervention, respectively, based on approach type and reflux type. Figure 1 illustrates surgical outcomes by VUR grade in pediatric patients.

### 3.1. Patient Selection and Cohort Characteristics

A total of 90 pediatric patients with high-grade vesicoureteral reflux (VUR) underwent ureteral reimplantation between January 2019 and January 2024 and were included in the analysis. All patients had Grade III, IV, or V VUR confirmed by voiding cystourethrography (VCUG). The cohort was restricted to surgically treated patients; therefore, children with Grade III–V VUR who were managed conservatively during the same period were not included. Accordingly, these results describe outcomes among operated children and should not be interpreted as representing the full population of children with high-grade VUR or as a comparison between surgical and non-surgical management. Of the 90 included patients, 59 underwent open ureteral reimplantation, and 31 underwent laparoscopic ureteral reimplantation. Open surgery accounted for 65.6% of procedures, whereas laparoscopy accounted for 34.4%. The surgical approach was not randomly assigned. Open reimplantation was more frequently performed because it was consistently available and technically established in the institutional setting. Laparoscopic reimplantation was performed in selected patients when pediatric laparoscopic equipment, trained personnel, operating room conditions, and surgeon judgment supported a minimally invasive approach. No pneumo-vesicoscopic reimplantation was documented as a separate operative category in the analyzed dataset.

### 3.2. Demographic Characteristics

The cohort included 48 girls (53.3%) and 42 boys (46.7%). Half of the patients were older than 6 years at the time of surgery. Age distribution was as follows: 2 patients (2.2%) were aged 0–6 months, 9 patients (10.0%) were aged 6–12 months, 16 patients (17.8%) were aged 1–3 years, 18 patients (20.0%) were aged 3–6 years, and 45 patients (50.0%) were older than 6 years. Most patients were mestizo (79/90, 87.8%), followed by Afro-Ecuadorian patients (9/90, 10.0%), Native American patients (1/90, 1.1%), and patients from other ethnic groups (1/90, 1.1%). Regarding region of residence, 52 patients (57.8%) came from the Highlands, 33 (36.7%) from the Coast, and 5 (5.6%) from the Amazon region. No statistically significant differences were observed between the open and laparoscopic groups according to sex (*p* = 0.123), age group (*p* = 0.313), ethnicity (*p* = 0.406), or region of residence (*p* = 0.427).

### 3.3. Preoperative Clinical Characteristics

Most children were born at term. Gestational age was 37–41 weeks in 65 patients (72.2%), 32–37 weeks in 18 patients (20.0%), and more than 41 weeks in 7 patients (7.8%). Prenatal ultrasound showed mild hydronephrosis in 56 patients (62.2%), severe hydronephrosis in 28 patients (31.1%), and normal findings in 6 patients (6.7%). A history of febrile urinary tract infection was documented in 62 patients (68.9%), and dysuria was reported in 23 patients (25.6%). These preoperative clinical characteristics did not differ significantly between patients treated with open surgery and those treated laparoscopically, including gestational age (*p* = 0.204), prenatal ultrasound findings (*p* = 0.985), febrile urinary tract infection (*p* = 0.205), and dysuria (*p* = 0.639).

### 3.4. VUR Grade, Laterality, and Imaging Findings

Reflux severity was classified by VCUG grade. Grade III VUR was present in 41 patients (45.6%), Grade IV in 39 patients (43.3%), and Grade V in 10 patients (11.1%). Reflux laterality was left-sided in 34 patients (37.8%), right-sided in 41 patients (45.6%), and bilateral in 15 patients (16.7%). The distribution of VUR grade did not differ significantly between surgical approaches (*p* = 0.778), and reflux laterality was also similar between groups (*p* = 0.309). Renal ultrasound data were available for 88 patients. Among these, renal ultrasound was abnormal in 72 patients (81.8%) and normal in 16 patients (18.2%). VCUG was abnormal in all included patients, consistent with the inclusion criterion requiring confirmed Grade III–V VUR. Preoperative DMSA scintigraphy, differential renal function, renal scarring, bladder dysfunction, and VUR etiology were reviewed when available. However, these variables were not uniformly documented across all records. Because documentation of VUR etiology and bladder dysfunction was incomplete, postoperative outcomes were not stratified by primary versus secondary VUR in the main analysis. The source manuscript does not provide complete variable-level missingness counts for DMSA findings, renal scarring, renal function, bladder dysfunction, VUR etiology, surgeon experience, or follow-up duration.

### 3.5. Missing Data and Follow-Up Availability

No statistical imputation was performed, and analyses were conducted using available-case denominators. Renal ultrasound data were available for 88 of 90 patients (97.8%), with 2 patients (2.2%) lacking ultrasound classification. VCUG data were available for all 90 patients (100%) because abnormal VCUG-confirmed Grade III–V VUR was required for inclusion. Surgical approach, VUR grade, reflux laterality, operative time category, intraoperative complication category, reflux resolution, recurrence, and reintervention were available for all included patients.

Several clinically important variables were incompletely and non-uniformly documented in the retrospective record, including VUR etiology, bladder-bowel dysfunction, DMSA scintigraphy findings, renal scarring, differential renal function, surgeon experience, detailed operative technique, follow-up duration, and the method used to confirm reflux resolution. Because these variables were not recorded in a standardized manner, valid variable-level missingness estimates could not be calculated. They were therefore not included in adjusted models.

Follow-up duration was not uniformly documented and could not be summarized using a reliable median, interquartile range, or range. Therefore, recurrence and reintervention should be interpreted as crude postoperative event proportions rather than time-adjusted outcome rates. This limitation is clinically relevant because reflux resolution, recurrence, and reintervention are time-dependent outcomes, and unequal follow-up may have introduced informative censoring.

Among the 68 patients classified as having reflux resolution, the available retrospective records did not consistently distinguish imaging-confirmed resolution from resolution based on explicit pediatric urologist documentation. Therefore, the proportions of imaging-confirmed resolution and clinically documented resolution could not be reliably calculated. This limitation should be considered when interpreting the overall resolution rate of 75.6%, because imaging-confirmed resolution and specialist-documented resolution are not equivalent outcome ascertainment standards.

### 3.6. Surgical Approach and Operative Characteristics

Surgical laterality was left-sided in 35 patients (38.9%), right-sided in 39 patients (43.3%), and bilateral in 16 patients (17.8%). By surgical approach, left-sided repair was performed in 13 laparoscopic cases and 22 open cases, right-sided repair in 15 laparoscopic cases and 24 open cases, and bilateral repair in 3 laparoscopic cases and 13 open cases. Surgical laterality did not differ significantly between groups (*p* = 0.344).

Open ureteral reimplantation was performed in 59 patients (65.6%), and laparoscopic ureteral reimplantation was performed in 31 patients (34.4%). Operative time differed significantly between groups. Surgery lasted 2–3 h in 34 patients overall, including 32 open cases (54.2% of open procedures) and 2 laparoscopic cases (6.5% of laparoscopic procedures). Surgery lasted 3–4 h in 32 patients overall, including 17 open cases (28.8%) and 15 laparoscopic cases (48.4%). Surgery lasted more than 4 h in 24 patients overall, including 10 open cases (16.9%) and 14 laparoscopic cases (45.2%). The distribution of operative time differed significantly between surgical approaches (*p* < 0.001). Continuous operative time values, including mean operative time and range by approach, were not available in the source manuscript; therefore, only categorical operative time can be reported from the provided dataset.

### 3.7. Intraoperative Complications

Intraoperative complications were documented in 20 patients (22.2%), whereas 70 patients (77.8%) had no recorded intraoperative complications. No complication was recorded in 49 of 59 open cases (83.1%) and in 21 of 31 laparoscopic cases (67.7%). Technical conversion occurred in 3 patients (3.3% overall), all in the laparoscopic group, representing 9.7% of laparoscopic cases. Bladder injury occurred in 2 patients (2.2% overall), including 1 open case (1.7%) and 1 laparoscopic case (3.2%). Other intraoperative events were recorded in 15 patients (16.7% overall), including 9 open cases (15.3%) and 6 laparoscopic cases (19.4%). The overall distribution of intraoperative complications did not differ significantly between open and laparoscopic surgery (*p* = 0.081). The source manuscript does not provide a detailed breakdown of the 15 events classified as “other” intraoperative complications. It also does not specify the exact mechanism of each technical conversion. Therefore, the Results section can report the documented categories—technical conversion, bladder injury, and other events—but cannot further subdivide “other” complications without access to the original operative records. The three technical conversions occurred only in the laparoscopic group and were described in the manuscript as related mainly to technical or equipment-related limitations; however, the specific limitations are not individually itemized in the source text.

### 3.8. Overall Postoperative Outcomes

Overall, reflux resolution was achieved in 68 of 90 patients (75.6%). Reflux recurrence occurred in 12 patients (13.3%), and reintervention was required in 8 patients (8.9%). Resolution was achieved in 44 of 59 patients treated with open surgery (74.6%) and in 24 of 31 patients treated laparoscopically (77.4%) (*p* = 0.766). Recurrence was observed in 10 of 59 open cases (16.9%) and in 2 of 31 laparoscopic cases (6.5%) (*p* = 0.164). Reintervention occurred in 5 of 59 open cases (8.5%) and in 3 of 31 laparoscopic cases (9.7%) (*p* = 0.849). Thus, open and laparoscopic reimplantation showed similar resolution and reintervention rates in this surgically selected cohort, while recurrence was numerically lower after laparoscopy without reaching statistical significance. Among the 68 patients classified as having reflux resolution, the source manuscript does not report how many were confirmed by postoperative imaging and how many were classified by explicit pediatric urologist documentation without confirmatory postoperative VCUG. Therefore, the proportion of imaging-confirmed resolution versus clinically documented resolution cannot be calculated from the provided manuscript. This absence should be stated explicitly because mixed outcome ascertainment may influence the apparent resolution rate.

### 3.9. Outcomes According to VUR Grade

Postoperative outcomes varied according to VUR grade. Resolution was highest among patients with Grade III VUR, intermediate among patients with Grade IV VUR, and lowest among patients with Grade V VUR. Conversely, recurrence and reintervention increased with reflux severity. Grade V VUR was present in 10 patients, representing 11.1% of the cohort. Within this small subgroup, reflux resolution occurred in 2 of 10 patients (20.0%), recurrence occurred in 4 of 10 patients (40.0%), and reintervention was required in 3 of 10 patients (30.0%).

These absolute event counts are important for contextualizing the exploratory regression estimates. Although Grade V VUR was associated with lower odds of resolution and higher odds of recurrence compared with Grade III VUR, the Grade V subgroup included only 10 patients. Therefore, the corresponding odds ratios should be interpreted cautiously because a small number of events can generate unstable estimates and wide confidence intervals. The findings support an association between higher reflux grade and less favorable postoperative outcomes, but they do not establish reflux grade as an independent or causal determinant of prognosis.

### 3.10. Exploratory Regression Analysis of Reflux Resolution

An exploratory logistic regression model was used to examine measured factors associated with reflux resolution. The model included sex, age group, surgical approach, and VUR grade. Compared with Grade III VUR, Grade V VUR was associated with lower odds of reflux resolution (odds ratio [OR], 0.06; 95% CI, 0.01–0.40; *p* = 0.003). Grade IV VUR showed a non-significant trend toward lower odds of resolution (OR, 0.31; 95% CI, 0.09–1.05; *p* = 0.060). Surgical approach was not independently associated with reflux resolution (laparoscopic vs. open: OR, 1.44; 95% CI, 0.45–4.64; *p* = 0.543). Sex and age group were also not significantly associated with resolution. These regression findings should be interpreted as exploratory. The model did not include several clinically important variables, including VUR etiology, bladder dysfunction, renal scarring, renal function, surgeon experience, operative technique details, and follow-up intensity, because these variables were incompletely documented or not suitable for inclusion given the study size and event distribution.

### 3.11. Exploratory Regression Analysis of Reflux Recurrence

A separate exploratory logistic regression model was used to examine measured factors associated with reflux recurrence. Twelve recurrence events were observed in the full cohort. Grade V VUR was associated with higher odds of recurrence compared with Grade III VUR (OR, 16.69; 95% CI, 1.88–148.32; *p* = 0.012). Grade IV VUR was not significantly associated with recurrence (OR, 2.48; 95% CI, 0.49–12.48; *p* = 0.270). Surgical approach was not statistically associated with recurrence (laparoscopic vs. open: OR, 0.22; 95% CI, 0.04–1.34; *p* = 0.101). Sex and age group were not statistically significant predictors of recurrence. Because only 12 recurrence events were available, this model is vulnerable to overfitting and statistical instability. The wide confidence interval for Grade V VUR indicates substantial imprecision, and the result should not be interpreted as a definitive or causal estimate. The absolute number of recurrence events in the Grade V subgroup was not reported in the source manuscript. Therefore, the regression result should be contextualized by stating that the Grade V subgroup included only 10 patients and that the exact number of recurrence events within this subgroup was unavailable in the manuscript.

### 3.12. Exploratory Regression Analysis of Reintervention

Reintervention was required in 8 patients. In the exploratory logistic regression model, no variable was statistically associated with the need for reintervention. Grade IV VUR showed a non-significant trend toward higher odds of reintervention compared with Grade III VUR (OR, 7.19; 95% CI, 0.78–66.23; *p* = 0.082). Grade V VUR also showed a non-significant trend toward higher odds of reintervention (OR, 7.65; 95% CI, 0.56–104.09; *p* = 0.127). Surgical approach was not associated with reintervention (laparoscopic vs. open: OR, 2.19; 95% CI, 0.44–11.02; *p* = 0.340). Sex and age group were not associated with reintervention in this model. Because only 8 reintervention events occurred, this analysis should be considered descriptive and hypothesis-generating rather than confirmatory. The absolute number of reinterventions in the Grade V subgroup was not reported in the source manuscript. Given the limited number of events and the small Grade V subgroup, the regression model should not be used to infer the absence or presence of a definitive association between surgical approach, VUR grade, and reintervention.

In this surgically treated cohort of 90 Ecuadorian children with Grade III–V VUR, overall reflux resolution was achieved in 75.6% of patients, recurrence occurred in 13.3%, and reintervention was required in 8.9%. Resolution rates were similar between open and laparoscopic surgery, at 74.6% and 77.4%, respectively. Laparoscopic surgery required longer operative times than open surgery, with 45.2% of laparoscopic cases lasting more than 4 h compared with 16.9% of open cases. Technical conversion occurred only in laparoscopic cases. Outcomes appeared less favorable among patients with Grade V VUR, but this subgroup included only 10 patients, and the absolute number of Grade V recurrence and reintervention events was not provided in the source manuscript. Therefore, the observed associations between VUR grade and postoperative outcomes should be interpreted as exploratory and potentially affected by selection bias, incomplete documentation of reflux etiology and bladder dysfunction, non-standardized follow-up, and limited event numbers.

## 4. Discussion

This retrospective cohort study evaluated postoperative outcomes among Ecuadorian children who underwent ureteral reimplantation for Grade III–V vesicoureteral reflux (VUR) at a tertiary pediatric referral center. Overall reflux resolution was achieved in 75.6% of patients, recurrence occurred in 13.3%, and reintervention was required in 8.9%. Open and laparoscopic reimplantation showed similar resolution and reintervention rates, whereas recurrence was numerically lower after laparoscopy but did not reach statistical significance. Higher VUR grade, particularly Grade V disease, was associated with less favorable postoperative outcomes in exploratory analyses. However, these findings should be interpreted as associations observed in a surgically selected cohort, not as evidence that reflux grade independently determines postoperative prognosis.

The observed relationship between higher reflux grade and poorer postoperative outcomes is clinically plausible because increasing anatomical severity may reflect greater ureterovesical junction incompetence, upper tract dilation, abnormal ureteral anatomy, renal injury, or more complex clinical disease. In this cohort, Grade V VUR showed the least favorable postoperative profile, with lower resolution and higher recurrence in the exploratory regression model. Nevertheless, only 10 patients had Grade V VUR, and the model for recurrence was based on only 12 events in the entire cohort. The odds ratio for Grade V recurrence, therefore, has a very wide confidence interval and should not be interpreted as a precise or definitive estimate. These results support the use of reflux grade as one marker of postoperative risk, but they do not establish reflux severity as the only or dominant determinant of outcome.

A central limitation is the incomplete distinction between primary and secondary VUR. This distinction is clinically essential because primary VUR, usually related to congenital incompetence of the ureterovesical junction, differs substantially from secondary VUR caused by posterior urethral valves, neurogenic bladder, bladder-bowel dysfunction, dysfunctional voiding, obstructive uropathy, or elevated bladder pressure. These etiologies may influence surgical indication, operative planning, recurrence, reintervention, and long-term renal function. In this study, reflux etiology and associated diagnoses were reviewed when available, but documentation was not sufficiently complete to support reliable stratified analyses. Consequently, some of the apparent association between VUR grade and outcome may reflect unmeasured differences in etiology, bladder dynamics, renal status, or anatomical complexity rather than reflux grade alone.

The comparison between open and laparoscopic reimplantation also requires cautious interpretation. The cohort included only surgically treated patients, and children with Grade III–V VUR managed conservatively during the same period were not included. In addition, the choice of surgical approach was not randomized. Open surgery was more frequently performed because it was widely available and technically established in the institutional setting, whereas laparoscopic surgery was used in selected patients when equipment, trained personnel, operating room conditions, and surgeon judgment allowed a minimally invasive approach. Therefore, comparison between approaches may be affected by confounding by indication. More complex cases may have been directed preferentially toward one approach, whereas equipment availability and institutional experience may have limited the use of laparoscopy in otherwise eligible patients.

Operative time was significantly longer in the laparoscopic group. Nearly half of laparoscopic procedures lasted more than 4 h, compared with a smaller proportion of open procedures. This finding should not be interpreted only as a technical disadvantage of laparoscopy; it may also reflect early institutional experience, the learning curve associated with pediatric minimally invasive reconstruction, case selection, anatomical complexity, equipment constraints, or workflow limitations. Longer operative times in children may have implications for anesthetic exposure, intraoperative risk, postoperative recovery, and resource utilization. Because surgeon-level experience, calendar period of laparoscopic adoption, case complexity, and continuous operative time values were not completely documented, the study could not determine whether longer laparoscopic operative time was related primarily to technique, learning curve, patient selection, or institutional resource constraints.

Intraoperative complications were documented in 20 patients. The recorded categories included technical conversion in 3 patients, bladder injury in 2 patients, and other intraoperative events in 15 patients. Technical conversion occurred only in the laparoscopic group. The manuscript indicates that conversions were mainly related to technical or equipment-related limitations, but the available source data do not provide a detailed case-by-case description of these limitations. Potential contributors include inadequate exposure, difficulty completing ureteral mobilization or tunneling safely, anatomical complexity, limitations of pediatric laparoscopic instruments, or operating room resource constraints. Because the “other” complication category was not further specified in the available dataset, the complication profile should be interpreted cautiously and should be improved in future studies through standardized operative reporting.

The primary outcome definition also introduces uncertainty. Reflux resolution was defined as the absence of VUR on postoperative imaging, preferably VCUG, or clear documentation by the treating pediatric urologist that reflux had resolved based on available follow-up assessment. These two standards are not equivalent. Imaging-confirmed resolution provides more objective evidence, whereas clinician documentation without confirmatory imaging may be influenced by incomplete notes, variable follow-up practices, symptoms, ultrasound findings, or clinical judgment. The source manuscript does not report the proportion of resolved cases confirmed by imaging versus clinical documentation alone. This mixed outcome definition may have inflated the apparent resolution rate and limits comparison with studies that require postoperative VCUG confirmation.

Follow-up duration is another important limitation because reflux resolution, recurrence, and reintervention are time-dependent outcomes. A patient followed for a few months and a patient followed for several years do not have the same opportunity to develop documented recurrence or require reintervention. The source manuscript indicates that follow-up duration was not uniformly documented and does not provide a complete median, interquartile range, or range. Therefore, the recurrence rate of 13.3% and reintervention rate of 8.9% should be interpreted in the context of incomplete and potentially unequal follow-up. Differential follow-up may have introduced informative censoring, particularly if patients with symptoms, complications, limited access to care, or more severe disease were more or less likely to return for postoperative assessment.

The exploratory regression analyses are limited by the small number of outcome events. Only 12 recurrence events and 8 reintervention events occurred, yet the models included several predictor blocks. This creates a substantial risk of overfitting and unstable estimates, especially for subgroup effects such as Grade V VUR. The wide confidence intervals around the recurrence and reintervention estimates reflect this imprecision. For this reason, the regression outputs should be viewed as hypothesis-generating and should not be used to support causal conclusions or definitive clinical predictions. The most reliable interpretation is descriptive: poorer outcomes appeared more frequent in patients with higher-grade reflux, but the magnitude and independence of this association remain uncertain.

Missing data further weakens causal and comparative interpretation. Relevant variables such as reflux etiology, bladder-bowel dysfunction, DMSA findings, renal scarring, renal function, antibiotic prophylaxis adherence, surgeon experience, operative technique details, socioeconomic status, follow-up duration, and imaging-confirmed resolution were incompletely documented. Available-case analysis assumes that missingness does not substantially distort associations, but this assumption may not hold in retrospective clinical records. For example, children with more complex diseases may have more detailed imaging, more frequent follow-up, or more complete documentation, while patients with limited access to care may have incomplete postoperative data. The direction of bias is therefore difficult to predict, and the true effect of missingness may be larger than the measured associations.

Missing and non-standardized documentation further limits interpretation. Several clinically relevant variables, including VUR etiology, bladder-bowel dysfunction, DMSA findings, renal scarring, differential renal function, surgeon experience, detailed operative technique, follow-up duration, and the method used to confirm reflux resolution, were reviewed when available but were not recorded in a standardized manner that allowed valid variable-level missingness estimates. For this reason, these variables were not included in adjusted models. Their incomplete documentation may have introduced residual confounding and information bias, and the direction of this bias cannot be reliably determined from the available retrospective records.

The study’s context is clinically important. As a tertiary pediatric referral center in Ecuador, the institution receives children from diverse geographic regions, including urban and rural areas. In this setting, diagnostic timing, referral pathways, access to pediatric subspecialists, imaging availability, operating room resources, minimally invasive equipment, and follow-up adherence may all influence observed outcomes. These system-level factors are not merely background characteristics; they may shape which patients reach surgery, which surgical approach is feasible, and how postoperative outcomes are documented. Therefore, the contribution of this study is not only the comparison of reflux grade or surgical approach, but also the description of real-world VUR care in an underrepresented middle-income healthcare setting.

These findings have practical implications. Children with Grade V VUR may require careful preoperative counseling and closer postoperative surveillance, but counseling should emphasize uncertainty and the influence of etiology, bladder function, renal status, and follow-up intensity. Comparisons between open and laparoscopic reimplantation should not be interpreted as evidence of superiority or equivalence, because allocation was nonrandomized and potentially influenced by clinical complexity and resource availability. Institutions performing pediatric laparoscopic reimplantation should document case selection, surgeon experience, operative route, equipment limitations, conversion criteria, operative time, complications, and longitudinal outcomes to better characterize the learning curve and safety profile of minimally invasive reconstruction.

This study has several limitations. First, the retrospective single-center design limits generalizability and precludes causal inference. Second, the cohort included only surgically treated patients, which creates selection bias and limits applicability to the broader VUR population. Third, the surgical approach was not randomly assigned, so comparisons between open and laparoscopic reimplantation are vulnerable to confounding by indication. Fourth, reflux etiology and bladder dysfunction were not sufficiently documented to permit reliable stratification of primary and secondary VUR. Fifth, the primary outcome combined imaging-confirmed resolution with clinician-documented resolution, which may have introduced outcome misclassification. Sixth, follow-up duration was incompletely documented, limiting the interpretation of recurrence and reintervention. Seventh, missingness in DMSA findings, renal function, surgeon experience, operative technique, and postoperative imaging may have introduced information bias. Finally, the small number of Grade V patients and the limited number of recurrence and reintervention events make regression estimates statistically unstable.

Despite these limitations, the study provides useful real-world data from an underrepresented pediatric population. Pediatric VUR outcomes from Latin American public referral systems remain insufficiently represented in the literature, and local data are important for understanding how healthcare access, imaging availability, referral patterns, surgical resources, and institutional capacity affect care delivery. The findings support the need for standardized documentation of reflux etiology, bladder-bowel dysfunction, renal imaging, DMSA findings, renal function, surgical indication, operative technique, surgeon experience, follow-up duration, postoperative imaging, and long-term renal outcomes. Future prospective multicenter studies should include conservatively managed, endoscopically treated, open, laparoscopic, and robotic cohorts; standardized definitions of resolution and recurrence; sufficient event numbers for multivariable modeling; and time-to-event analyses that account for follow-up duration.

## 5. Conclusions

In this retrospective cohort of surgically treated Ecuadorian children with Grade III–V vesicoureteral reflux, ureteral reimplantation was associated with reflux resolution in approximately three-quarters of patients. Open and laparoscopic reimplantation showed similar resolution and reintervention rates, while recurrence was numerically lower after laparoscopy but did not reach statistical significance. Higher reflux grade, particularly Grade V disease, was associated with less favorable postoperative outcomes in exploratory analyses; however, this finding should be interpreted cautiously because the Grade V subgroup was small, recurrence and reintervention events were few, and several important prognostic variables were incompletely documented.

These results support the clinical value of considering reflux grade during postoperative risk stratification, but they do not establish reflux severity as an independent or causal determinant of surgical outcome. Reflux etiology, bladder dysfunction, renal scarring, renal function, operative technique, surgeon experience, access to minimally invasive resources, and follow-up intensity may all influence postoperative resolution, recurrence, and reintervention. Because the cohort included only operated patients, the findings apply to a selected surgical population and should not be generalized to all children with high-grade VUR.

In middle-income healthcare settings, real-world outcomes after pediatric ureteral reimplantation may be shaped by referral timing, imaging availability, institutional surgical resources, equipment access, and continuity of postoperative follow-up. Future prospective multicenter studies should use standardized definitions of reflux resolution and recurrence, distinguish imaging-confirmed from clinically documented outcomes, report follow-up duration, include a complete assessment of primary and secondary VUR, and ensure adequate event numbers for robust multivariable analysis. Such studies are needed to clarify the relative contribution of reflux grade, etiology, bladder function, surgical approach, and healthcare access to long-term renal and surgical outcomes.

## Figures and Tables

**Table 1:** Distribution of patients by therapeutic management according to demographic characteristics.

Demography	Total	Surgery		*p*-Value
		Open Surgery	Laparoscopic	
Sex (n (%))				
Men	42 (46.67)	31 (52.54)	11 (35.48)	0.123
Women	48 (53.33)	28 (47.46)	20 (64.52)	
Group age (n (%))				
0–6 months	2 (2.22)	2 (3.39)	0 (0)	0.313
6–12 months	9 (10)	7 (11.86)	2 (6.45)	
1–3 years	16 (17.78)	13 (22.03)	3 (9.68)	
3–6 years	18 (20)	11 (18.64)	7 (22.58)	
>6 years	45 (50)	26 (44.07)	19 (61.29)	
Ethnic group (n (%))				
Mestizo	79 (87.78)	53 (89.83)	26 (83.87)	0.406
Afroecuadorian	9 (10)	5 (8.47)	4 (12.9)	
Native American	1 (1.11)	0 (0)	1 (3.23)	
Other	1 (1.11)	1 (1.69)	0 (0)	
Region of residence (n (%))				
Coast	33 (36.67)	19 (32.2)	14 (45.16)	0.427
Highlands	52 (57.78)	36 (61.02)	16 (51.61)	
Amazonia	5 (5.56)	4 (6.78)	1 (3.23)	

Note: Based on the Chi-square test; Source: Participating hospitals; prepared by the authors.

**Table 2:** Distribution of patients by therapeutic management according to clinical characteristics.

Clinical Findings	Total	Surgery		*p*-Value
		Open Surgery	Laparoscopic	
Gestational age (n (%)) in weeks				
32–37	18 (20)	10 (16.95)	8 (25.81)	0.204
37–41	65 (72.22)	46 (77.97)	19 (61.29)	
>41	7 (7.78)	3 (5.08)	4 (12.9)	
Prenatal ultrasound diagnosis (n (%))				
Mild hydronephrosis	56 (62.22)	37 (62.71)	19 (61.29)	0.985
Severe hydronephrosis	28 (31.11)	18 (30.51)	10 (32.26)	
Normal	6 (6.67)	4 (6.78)	2 (6.45)	
VUR grade (n (%))				
III	41 (45.56)	28 (47.46)	13 (41.94)	0.778
IV	39 (43.33)	24 (40.68)	15 (48.39)	
V	10 (11.11)	7 (11.86)	3 (9.68)	
Laterality of reflux (n (%))				
Left	34 (37.78)	23 (38.98)	11 (35.48)	0.309
Right	41 (45.56)	24 (40.68)	17 (54.84)	
Bilateral	15 (16.67)	12 (20.34)	3 (9.68)	
Febrile UTI (n (%))	62 (68.89)	38 (64.41)	24 (77.42)	0.205
Dysuria (n (%))	23 (25.56)	16 (27.12)	7 (22.58)	0.639
Radiology findings				
Renal ultrasound (n (%))				
Normal	16 (18.18)	10 (17.54)	6 (19.35)	0.833
Abnormal	72 (81.82)	47 (82.46)	25 (80.65)	
Voiding cystourethrography (n (%))				
Normal	0 (0)	0 (0)	0 (0)	1.000
Abnormal	90 (100)	59 (100)	31 (100)	

Note: Based on the Chi-square test; Source: Participating hospitals; prepared by the authors.

**Table 3:** Distribution of patients by therapeutic management according to characteristics of the surgery.

Surgery Description	Total	Surgery		*p*-Value
		Open Surgery	Laparoscopic	
Age at surgery (n (%))				
0–6 months	2 (2.22)	2 (3.39)	0 (0)	0.303
6–12 months	8 (8.89)	6 (10.17)	2 (6.45)	
1–3 years	17 (18.89)	14 (23.73)	3 (9.68)	
3–6 years	18 (20)	11 (18.64)	7 (22.58)	
>6 years	45 (50)	26 (44.07)	19 (61.29)	
Laterality of the surgical approach (n (%))				
Left	35 (38.9)	22 (37.3)	13 (41.9)	0.344
Right	39 (43.3)	24 (40.7)	15 (48.4)	
Bilateral	16 (17.8)	13 (22)	3 (9.7)	
Surgery time (n (%)) in hours				
2–3	34 (37.8)	32 (54.2)	2 (6.5)	<0.001 *
3–4	32 (35.6)	17 (28.8)	15 (48.4)	
>4	24 (26.7)	10 (16.9)	14 (45.2)	
Intraoperative complications (n (%))				
None	70 (77.78)	49 (83.05)	21 (67.74)	0.081
Technical Conversion	3 (3.33)	0 (0)	3 (9.68)	
Bladder Injury	2 (2.22)	1 (1.69)	1 (3.23)	
Other	15 (16.67)	9 (15.25)	6 (19.35)	
Postoperative outcome (n (%))				
Reflux resolution	68 (75.56)	44 (74.58)	24 (77.42)	0.766
Reflux recurrence	12 (13.33)	10 (16.95)	2 (6.45)	0.164
Reintervention	8 (8.89)	5 (8.47)	3 (9.68)	0.849

Note: Based on the Chi-square test; Source: Participating hospitals; prepared by the authors. * Significance.

**Table 4:** Multivariate relationship to predict **resolved reflux** based on approach type and reflux type.

Variables	B	Wald	*p*-Value	Odds Ratio (OR)	95% CI for OR	
					Lower	Upper
Sex						
Men(reference)						
Women	−0.17	0.09	0.770	0.85	0.27	2.61
Age groups						
0–12 months (reference)						
1–3 y	−1.95	2.33	0.127	0.14	0.01	1.74
3–6 y	−1.53	1.39	0.239	0.22	0.02	2.76
>6 y	−1.91	2.37	0.123	0.15	0.01	1.68
Type of surgical approach						
Open surgery (reference)						
Laparoscopic	0.36	0.37	0.543	1.44	0.45	4.64
VUR grade						
III (reference)						
IV	−1.18	3.54	0.060	0.31	0.09	1.05
V	−2.75	8.66	0.003	0.06	0.01	0.40

Note: Logistic regression model. OR, odds ratio; CI, confidence interval. Estimates should be interpreted cautiously because of the limited number of outcome events. Source: Participating hospitals; prepared by the authors.

**Table 5:** Multivariate relationship to predict **reflux recurrence** based on the type of approach and the type of reflux.

Variables	B	Wald	*p*-Value	Odds Ratio (OR)	95% CI for OR	
					Lower	Upper
Sex						
Men(reference)						
Women	0.29	0.15	0.694	1.33	0.32	5.56
Age groups						
0–12 months (reference)						
1–3 y	0.65	0.22	0.640	1.92	0.12	29.69
3–6 y	0.87	0.37	0.541	2.40	0.15	39.35
>6 y	1.48	1.23	0.267	4.39	0.32	59.60
Type of surgical approach						
Open surgery (reference)						
Laparoscopic	−1.51	2.69	0.101	0.22	0.04	1.34
VUR grade						
III (reference)						
IV	0.91	1.22	0.270	2.48	0.49	12.48
V	2.82	6.38	0.012 *	16.69	1.88	148.32

Note: Logistic regression model. OR, odds ratio; CI, confidence interval. Estimates should be interpreted cautiously because of the limited number of outcome events. Source: Participating hospitals; prepared by the authors. * Significance.

**Table 6:** Multivariate relationship to predict **reintervention** based on the type of approach and the type of reflux.

Variables	B	Wald	*p*-Value	Odds Ratio (OR)	95% CI for OR	
					Lower	Upper
Sex						
Men(reference)						
Women	−0.12	0.03	0.874	0.88	0.20	4.01
Age groups						
0–12 months (reference)						
1–3 y	0.96	0.56	0.456	2.60	0.21	32.17
3–6 y	0.15	0.01	0.911	1.16	0.08	16.31
>6 y	−0.41	0.10	0.753	0.67	0.05	8.38
Type of surgical approach						
Open surgery (reference)						
Laparoscopic	0.79	0.91	0.340	2.19	0.44	11.02
VUR grade						
III (reference)						
IV	1.97	3.03	0.082	7.19	0.78	66.23
V	2.03	2.33	0.127	7.65	0.56	104.09

Note: Logistic regression model. OR, odds ratio; CI, confidence interval. Estimates should be interpreted cautiously because of the limited number of outcome events. Source: Participating hospitals; prepared by the authors.

## Data Availability

The datasets used and analyzed during the current study are available from the corresponding author on reasonable request.

## Abbreviations

The following abbreviations are used in this manuscript:

CAP	continuous antibiotic prophylaxis
CI	confidence interval
CKD	chronic kidney disease
DMSA	dimercaptosuccinic acid
IQR	interquartile range
LMICs	low- and middle-income countries
OR	odds ratio
OU	open ureteral reimplantation
SPSS	Statistical Package for Social Sciences
STROBE	Strengthening the Reporting of Observational Studies in Epidemiology
UTI	urinary tract infection
VCUG	voiding cystourethrography
VUR	vesicoureteral reflux

## References

[1] Chang C.L., Yang S.S., Hsu C.K., Chen C.H., Chang S.J. (2023). Effectiveness of various treatment modalities in children with vesicoureteral reflux grades II–IV: A systematic review and network meta-analysis. BMJ Paediatr. Open.

[2] Miyakita H., Hayashi Y., Mitsui T., Okawada M., Kinoshita Y., Kimata T., Koikawa Y., Sakai K., Satoh H., Tokunaga M. (2020). Guidelines for the medical management of pediatric vesicoureteral reflux. Int. J. Urol..

[3] Hewitt I.K., Roebuck D.J., Montini G. (2023). Conflicting views of physicians and surgeons concerning pediatric urinary tract infection: A comparative review. Pediatr. Radiol..

[4] Gkalonaki I., Schoina E., Anastasakis M., Patoulias I. (2023). Pathogenesis and prognosis of intrarenal reflux. Folia Med. Cracov..

[5] Hwang J., Kim P.H., Yoon H.M., Song S.H., Jung A.Y., Lee J.S., Cho Y.A. (2023). Application of the postnatal urinary tract dilation classification system to predict the need for surgical intervention among neonates and young infants. Ultrasonography.

[6] Simões ESilva A.C., Oliveira E.A., Mak R.H. (2020). Urinary tract infection in pediatrics: An overview. J. Pediatr..

[7] Oukhouya M.A., Andaloussi S., Tazi M., Mahmoudi A., Khattala K., Bouabdallah Y. (2019). L’évolution à long terme du reflux vésico-rénal chez l’enfant [Long-term evolution of vesicoureteral reflux in children]. Pan. Afr. Med. J..

[8] Sforza S., Marco B.B., Haid B., Baydilli N., Donmez M.I., Spinoit A.F., Spinoit A.-F., Paraboschi I., Masieri L., Steinkellner L. (2024). A multi-institutional European comparative study of open versus robotic-assisted laparoscopic ureteral reimplantation in children with high grade (IV–V) vesicoureteral reflux. J. Pediatr. Urol..

[9] Ai J.W., Liu Y., Zeng X.T., Lei Q., Zou L., Pei B. (2015). Angiotensin Converting Enzyme Gene Insertion/Deletion Polymorphism and Vesicoureteral Reflux in Children: A Meta-Analysis of 14 Case-Control Studies. Medicine.

[10] Burki T., Howeiti M.S., Almadhi M.K., Al Modhen F.M., Alhazmi H., Vallasciani S.A., Alhams A.E., Mehmood S.W., Al Shammari A.M. (2020). Outcome of salvage ureteral reimplantation after endoscopic treatment failure for high-grade vesicoureteral reflux compared to primary ureteral reimplantation. Urol. Ann..

[11] Karabacak O.R., Yalçınkaya F., Altuğ U., Sertçelik N., Demirel F. (2014). Does the modıfıed STING method increase the success rate in the management of moderate or hıgh-grade reflux?. Korean J. Urol..

[12] Taşkinlar H., Avlan D., Bahadir G.B., Delibaş A., Nayci A. (2016). The outcomes of two different bulking agents (dextranomer hyaluronic acid copolymer and polyacrylate-polyalcohol copolymer) in the treatment of primary vesico-ureteral reflux. Int. Braz. J. Urol..

[13] Faiz S., Zaveri M.P., Perry J.C., Schuetz T.M., Cancarevic I. (2020). Role of Antibiotic Prophylaxis in the Management of Antenatal Hydronephrosis, Vesicoureteral Reflux, and Ureterocele in Infants. Cureus.

[14] Liang D., McHugh K.M., Brophy P.D., Shaikh N., Manak J.R., Andrews P., Hakker I., Wang Z., Schwaderer A.L., Hains D.S. (2019). DNA copy number variations in children with vesicoureteral reflux and urinary tract infections. PLoS ONE.

[15] Guler Y., Erbin A., Ozmerdiven G. (2020). Modified Lich-Gregoir Ureteral Reimplantation for the Treatment of Unilateral Primary Vesicoureteral Reflux in Pediatric Patients: A Comparative Analysis with Medium-Term outcomes. Urol. J..

[16] Amar A.D., Singer B., Chabra K. (1976). The practical management of vesicoureteral reflux in children. A review of 12 years’ experience with 236 patients. Clin. Pediatr..

[17] Keren R., Shaikh N., Pohl H., Gravens-Mueller L., Ivanova A., Zaoutis L., Patel M., deBerardinis R., Parker A., Bhatnagar S. (2015). Risk Factors for Recurrent Urinary Tract Infection and Renal Scarring. Pediatrics.

[18] Alvarez Garcia N., Siles Hinojosa A., Rihuete Heras M.A., Justa Roldan M., Delgado Alvira R., González Ruiz Y., Gracia Romero J., Fernández Atuan R. (2017). Impact of using an evidence-based clinical guideline for the management of primary vesicoureteral reflux in children. Impacto de la apación de una guía clínica basada en la evidencia en el tratamiento del reflujo vesicoureteral primario en el niño. Arch. Argent. Pediatr..

[19] Pensabene M., Cimador M., Spataro B., Serra G., Baldanza F., Grasso F., Corsello G., Salerno S., Di Pace M.R., Sergio M. (2024). Intraoperative ultrasound-assisted endoscopic treatment of primary intermediate and high-grade vesicoureteral reflux in children in a long-term follow-up. J. Pediatr. Urol..

[20] Pensabene M., Spataro B., Baldanza F., Grasso F., Serra G., Notarbartolo V., Giuffrè M., Corsello G., Zambaiti E., Di Pace M.R. (2025). Application of Ultrasound in Primary Vesicoureteral Reflux: From Diagnosis to Follow Up. Children.

[21] Peters C.A., Skoog S.J., Arant B.S., Copp H.L., Elder J.S., Hudson R.G., Khoury A.E., Lorenzo A.J., Pohl H.G., Shapiro E. (2010). Summary of the AUA Guideline on Management of Primary Vesicoureteral Reflux in Children. J. Urol..

[22] Johnston D.L., Qureshi A.H., Irvine R.W., Giel D.W., Hains D.S. (2016). Contemporary Management of Vesicoureteral Reflux. Curr. Treat. Options Pediatr..

[23] von Elm E., Altman D.G., Egger M., Pocock S.J., Gøtzsche P.C., Vandenbroucke J.P. (2008). The Strengthening the Reporting of Observational Studies in Epidemiology (STROBE) statement: Guidelines for reporting observational studies. J. Clin. Epidemiol..

[24] Peduzzi P., Concato J., Kemper E., Holford T.R., Feinstein A.R. (1996). A simulation study of the number of events per variable in logistic regression analysis. J. Clin. Epidemiol..

[25] Vittinghoff E., McCulloch C.E. (2007). Relaxing the rule of ten events per variable in logistic and Cox regression. Am. J. Epidemiol..

[26] Meara J.G., Leather A.J., Hagander L., Alkire B.C., Alonso N., Ameh E.A., Bickler S.W., Conteh L., Dare A.J., Davies J. (2015). Global Surgery 2030: Evidence and solutions for achieving health, welfare, and economic development. Surgery.

[27] Wilkinson E., Aruparayil N., Gnanaraj J., Brown J., Jayne D. (2021). Barriers to training in laparoscopic surgery in low- and middle-income countries: A systematic review. Trop. Doct..

[28] Buss R., SenthilKumar G., Bouchard M., Bowder A., Marquart J., Cooke-Barber J., Vore E., Beals D., Raval M., Rich B.S. (2022). Geographic barriers to children’s surgical care: A systematic review of existing evidence. J. Pediatr. Surg..

[29] Sterne J.A., White I.R., Carlin J.B., Spratt M., Royston P., Kenward M.G., Wood A.M., Carpenter J.R. (2009). Multiple imputation for missing data in epidemiological and clinical research: Potential and pitfalls. BMJ.

